# Sleep Quality among Police Officers: Implications and Insights from a Systematic Review and Meta-Analysis of the Literature

**DOI:** 10.3390/ijerph16050885

**Published:** 2019-03-11

**Authors:** Sergio Garbarino, Ottavia Guglielmi, Matteo Puntoni, Nicola Luigi Bragazzi, Nicola Magnavita

**Affiliations:** 1 Department of Neuroscience, Rehabilitation, Ophthalmology, Genetics and Maternal/Child Sciences, University of Genoa, Polyclinic Hospital San Martino IRCCS, 16132 Genoa, Italy; sgarbarino.neuro@gmail.com; 2 Clinical trial Office, E.O. Galliera Hospital, 16128 Genoa, Italy; matteo.puntoni@gmail.com; 3 UNESCO Chair of Anthropology of Health - Biosphere and Healing System, University of Genoa, 16132 Genoa, Italy; robertobragazzi@gmail.com; 4 Department of Woman/Child & Public Health, Università Cattolica del Sacro Cuore, Fondazione Policlinico Gemelli IRCCS, 00168 Roma, Italy; nicolamagnavita@gmail.com

**Keywords:** police, sleep, occupational health, meta-analysis, prevalence, public health, sleep deprivation, health promotion, sleep hygiene

## Abstract

Poor sleep is associated with bad health outcomes, worse wellbeing and decreases in performance, productivity and safety at work. Police officers are exposed to several risk factors including extended work schedules, shift work, occupational stress, dangerous and traumatic events and can, as such, develop sleep problems. The aim of the present study was to analyze the sleep quality among police officers. A systematic literature search, in PubMed/MEDLINE, PsycINFO, Scopus, ISI/Web of Science (WoS) and the Cochrane Library was conducted. Original articles, published in English, French, Italian, Spanish and Portuguese, the primary objective of which was the evaluation of the quality of sleep through the Pittsburgh Sleep Quality Index (PSQI) in Police Forces, were selected. From an initial sample of 1,149 studies, 13 articles were included in the meta-analysis (3,722 police officers). The pooled prevalence of bad sleep quality in police officers was 51% [95%CI 42–60%]. The pooled global score of the PSQI was 5.6 [95%CI 5.0–6.3], corresponding to a low average quality. At the meta-regressions, statistically significant associations could be found for work seniority (in terms of years of service) and being on shift. The poor quality of sleep in police officers could have negative consequences for workers’ health and for the safety of third parts. The implementation of health and sleep hygiene promotion programs in police forces is strongly recommended.

## 1. Introduction

Good sleep is important for maintaining general health and wellbeing, in that sleep is an integral component of human life and can impact on several variables and parameters of human physiology. Sleep disorders are common in the general population and affect up to one third of adults, both in high- and low-medium income countries [1]. Sleep problems are associated with bad health outcomes, and poor sleep quality has been shown to be associated with poor food choice, unhealthy dietary intakes [2], high body fat and obesity [3]. Deregulation of hypothalamic functions in sleep disorders can contribute to these alterations [4]. Bad sleep is a risk factor for metabolic syndrome [5] and impacts on lifestyle behaviors that affect cardiovascular health [6,7]. Bad sleepers are more exposed to mild cognitive impairment when compared to good sleepers [8]. For instance, Paunio et al. [9], in a longitudinal study in which a representative sample of the Finnish adult population was followed up for many decades, found that long-term poor sleep increased risk of depression and disability retirement due to depressive disorders. Furthermore, sleep disorders have been associated with negative work outcomes, poor health [10], work injuries [11] and driving accidents [12,13]. 

Many occupational factors can, indeed, alter workers’ sleep health. These include irregular shift work, night shift and extended work schedules [14]. In a recent meta-analysis, bad sleep quality was associated with an increased risk of work-related injuries [15]. Workers with poor sleep tend to report higher job stress than good sleepers [16] and some authors have observed that the bi-directionality of the relationships could indicate a vicious cycle, in which work-related stress, perseverative cognition, and sleep quality mutually influence each other [17]. Sleep may be also involved in the development of burnout, as individuals with symptoms of burnout tend to report sleep fragmentation [18], poorer subjective sleep quality and non-restorative sleep [19]. High levels of stress and fatigue are strictly inherent to police work. Police officers often experience extended work schedules, shift work, occupational stress, and dangerous and traumatic events [20,21]. Numerous studies have reported that, compared with other professions, police officers are at increased risk for stress-related physical illnesses, including heart disease and metabolic syndrome [22,23]. Furthermore, they are at high risk for developing psychological problems such as depression [24]. Rajaratnam et al. [25], in a study involving 4,957 police officers from two countries (the United States and Canada), observed a considerable percentage of sleep problems. A high prevalence of sleep disorders, excessive daytime sleepiness and sleep-related accidents has also been observed in Italian police officers [26]. Emotional traumas, shift work, and occupational stress interfere with the quality of sleep in police officers [27] and this can have significant consequences for workers’ health and public safety, hence the interest in systematically evaluating sleep in police forces. 

The aim of this study was to analyze sleep quality among police officers through a systematic review, appraisal and meta-analysis of the literature. In order to obtain fairly homogeneous data, so as to be able to summarize the results in a meta-analysis, we have limited our research to studies that have assessed the quality of sleep using the most used self-filled questionnaire, the Pittsburgh Sleep Quality Questionnaire (PSQI).

## 2. Materials and Methods 

### 2.1. Questionnaire

The PSQI [28] consists of 19 items and 7 clinically relevant domains of sleep difficulties: namely, i) subjective sleep quality, ii) sleep latency, iii) sleep duration, iv) sleep efficiency, v) sleep disturbances, vi) use of sleep medication, and vii) daytime dysfunction. A global score of overall sleep quality can be calculated by adding up the single scores of these dimensions, producing scores ranging from 0 to 21. 

Global scores >5 are generally used to indicate poor sleep. A recent meta-analysis has shown a strong reliability and validity, and a moderate structural validity of the PSQI in a variety of samples and settings, suggesting the tool fulfills its intended utility [29]. Given the PSQI’s widespread use as well as its psychometric soundness, we based our quantitative analysis on studies that adopted this tool.

### 2.2. Search Strategy

The present systematic review and meta-analysis has been conducted according to the “Preferred Reporting Items for Systematic reviews and Meta-Analyses” (PRISMA) guidelines. We systematically searched the following scholarly databases and bibliographic thesauri: namely, PubMed/MEDLINE, PsycINFO, Scopus, ISI/Web of Science (WoS) and the Cochrane Library from inception up to August 2018. Articles written in English, Italian, French, Spanish and Portuguese languages were considered for potential inclusion. 

The overall search strategy comprised of a string of keywords related to the profession under scrutiny (police OR police officer OR police force OR policeman OR policemen OR police operators) and sleep quality and its synonymous (sleep OR sleep disorders OR sleep quality OR Pittsburgh Sleep Quality Questionnaire OR PSQI), properly combined by Boolean operators. 

All the empirical studies (designed as cross-sectional, prospective, case-control or quasi-experimental investigations) were included. Studies focusing on sleep quality, assessed by the PSQI were selected. Studies that evaluated specific sleep disorders, or assessed sleep quality by means of tools different from the PSQI were excluded. Additionally, studies that used PSQI but lacked sufficient quantitative details—for instance, studies not reporting the PSQI global score or the prevalence rate of poor sleepers—were excluded. Finally, we excluded all secondary literature studies, such as reviews (of any type), systematic reviews and meta-analyses. These studies were, anyway, read and consulted for reducing the risk of missing investigations potentially relevant. In addition, we manually searched target journals and scanned reference lists of included studies for increasing the chance of getting articles related to the topic under study. 

### 2.3. Selection of Published Studies

First, two researchers (SG and OG) independently reviewed all titles/abstracts to identify potentially relevant articles. They, then, performed study selection, based on a full-text review, according to the previously stated inclusion/exclusion criteria. Disagreements were resolved by discussion with a third author (NM), acting as the final referee. Finally, selected studies meeting with pre-defined inclusion/exclusion criteria and coherent with the topic of interest were included in the present systematic review and meta-analysis. 

### 2.4. Study Quality Assessment

The assessment of the methodological quality of these studies was conducted applying the Newcastle–Ottawa scale (NOS) [30]. Disagreement on quality assessment and appraisal was resolved by discussion until consensus was reached. 

### 2.5. Data Extraction and Analysis

The following data were extracted for each study: study design, year of publication, sample size, country, mean age of police officers, percentage of female police officers, mean body mass index (BMI), smoking status, alcohol intake, marital status, education level (percentage of participants having completed high school), percentage of white and Hispanics participants, years of service, hierarchical role (sergeant, lieutenant, captain), percentage of police officers with administrative role, percentage of military police officers or with previous military experience, PSQI cut-off adopted for defining poor sleepers, prevalence rate of poor sleepers, mean PSQI global score, average sleep duration, and the main factors associated with sleep quality evaluated in the included studies. 

### 2.6. Quantitative Data Synthesis

The pooled prevalence of low/poor sleep quality was computed by the meta-analytic weighting of the original rates of police officers exceeding the PSQI cut-off adopted in each study. A further quantitative analysis was carried out pooling the PSQI global scores obtained in each study. Fixed-effect or DerSimonian–Laird random-effect models were applied according to the amount of heterogeneity found. Sensitivity analysis (removing each study per time) was carried out to investigate the stability and reliability of the findings. Univariate and multivariate meta-regressions were performed to assess the association between poor sleep quality/PSQI global score and the other variables under study.

In case of missing data, authors of the present systematic review and meta-analysis personally contacted the authors of the studies, who were requested to provide the missing values. For several articles, the proper parameters were computed by the authors themselves.

Heterogeneity among studies was assessed by visual inspection of the forest plot and by using a standard chi-squared test with a significance level of alpha = 0.01 [31]. The percentage of heterogeneity among the various studies was quantified by computing the I^2^ statistics, with I^2^ of 25%, 50% and 75% corresponding to a small, medium and large degree of heterogeneity, respectively [32]. 

Publication bias was evaluated by both visually inspecting the funnel plot and performing the Egger’s linear regression test. In case of evidence of publication bias, the “true” effect size (ES) was estimated by the trim-and-fill analysis. 

### 2.7. Statistical Software

All statistical analyses were performed with the commercial software Comprehensive Meta-Analysis (CMA version 3.0, for Windows, Biostat, Englewood, NJ, USA).

## 3. Results

**Figure 1:**
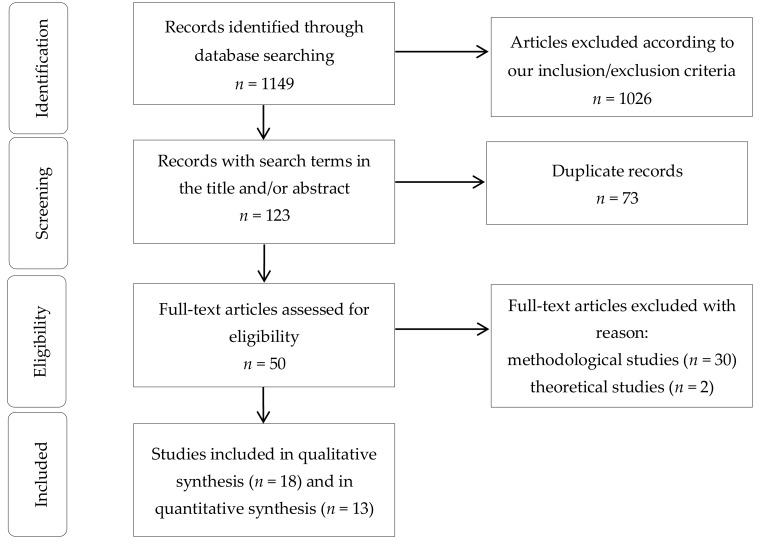
Article selection algorithm.

The initial search strategy produced a total of 1149 studies (Figure 1). 

We discarded 32 papers: 2 were theoretical investigations and 30 were methodological studies. 18 articles were included in the systematic review [33,34,35,36,37,38,39,40,41,42,43,44,45,46,47,48,49,50]. Furthermore, from the quantitative synthesis (i.e., the meta-analysis) several studies (*n* = 6) [36,40,48] were based on the same sample obtained from the “Buffalo Cardio-Metabolic Occupational Police Stress” (BCOPS) cohort trial and, as such, we included only the publication of Charles et al. (2011), having the largest sample size [36]. 

The final sample comprised a total of 3722 participants (Table 1), with a sample size that ranged from 22 [45] to 796 [35] police officers.

Most researches were conducted in the USA, two in Brazil [33,45], one in Australia [38], India [49], Iran [47], and China [35], respectively. All the studies included in the present systematic review and meta-analysis were cross-sectional. Overall, studies were characterized by medium-low quality according to the NOS. 

The heterogeneity among studies was high, exceeding 95% for both estimates (96.62% for the pooled PSQI global score and 95.86% for percentage of poor sleepers, respectively). Due to the large, statistically significant degree of heterogeneity, we calculated study weights and ES according to the DerSimonian–Laird random-effect model. 

**Figure 2:**
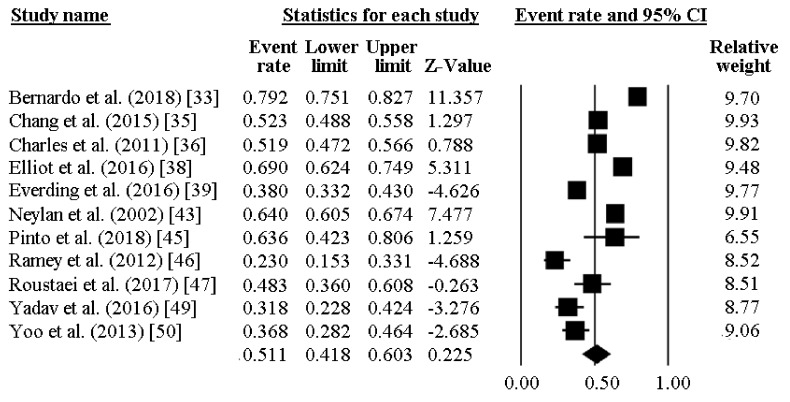
Prevalence of low sleep quality in police officers.

**Figure 3:**
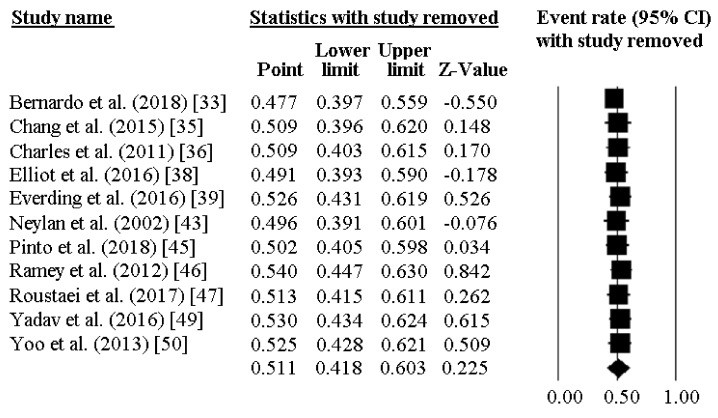
Sensitivity analysis for the prevalence of low sleep quality in police officers, showing stability and reliability of the findings.

**Figure 4:**
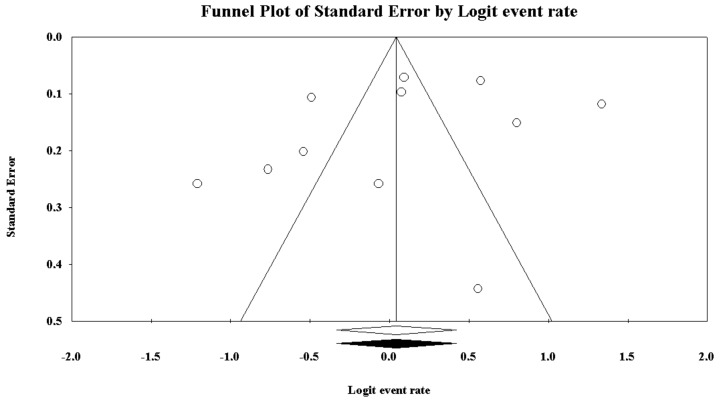
Funnel plot of the meta-analysis of the prevalence of low sleep quality in police officers.

The prevalence of poor sleep quality among police officers was reported by 11 investigations and ranged between 23% and 79%. As reported in Figure 2, the pooled prevalence of poor sleepers was 51.1% [95%CI 41.8–60.3]. Figure 3 reports the sensitivity analysis, showing the stability and reliability of the findings after removing each study per time. Finally, visual inspection of the funnel plot and computation of the Egger’s linear regression test did not reveal any evidence of publication bias regarding percentage of poor sleepers (Figure 4).

**Figure 5:**
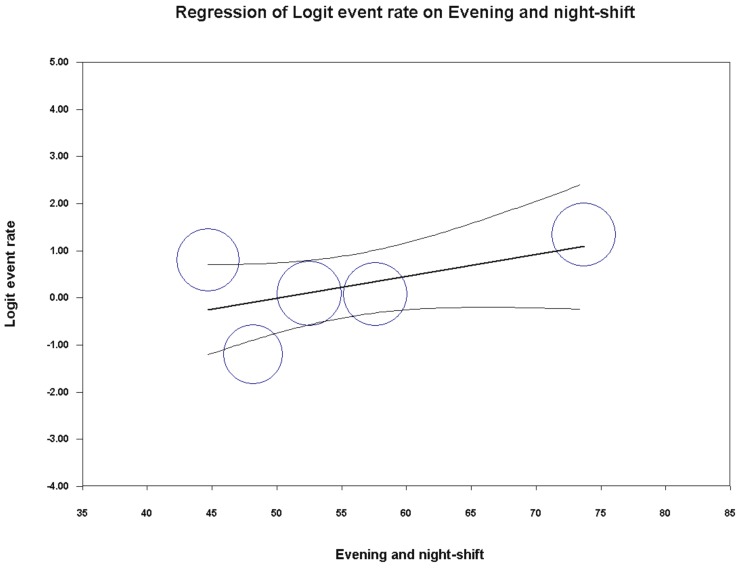
Meta-regression analysis showing a statistically borderline association between prevalence of low sleep quality in police officers and percentage of police officers on evening/night shift.

Univariate meta-regression analysis failed to capture any significant association between the percentage of poor sleepers and the variables under study (publication year *p* = 0.8762; country *p* = 0.1066; mean age *p* = 0.2252; percentage of female participants *p* = 0.9604; BMI *p* = 0.9844; hierarchical role *p* = 0.3549). Interestingly, the association between the percentage of poor sleeper and the policemen under evening/night shift yielded a statistically borderline result (coefficient = 0.05, SE = 0.03 [95%CI –0.01 to 0.10], z-value = 1.76, *p* = 0.0782), as shown in Figure 5. For the other variables under study, it was not possible to perform any meta-regression analysis due to the insufficient number of studies. Furthermore, for the same reason, it was not possible to run a multivariate meta-regression analysis.

**Figure 6:**
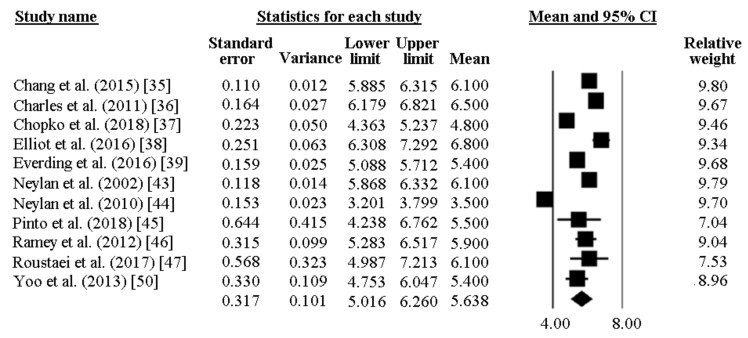
Pooled global score of PSQI in police officers.

**Figure 7:**
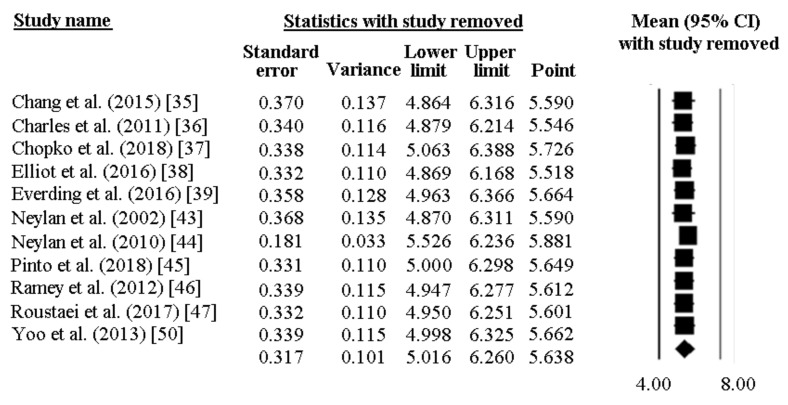
Sensitivity analysis for the pooled PSQI global score in police officers, showing stability and reliability of the findings.

**Figure 8:**
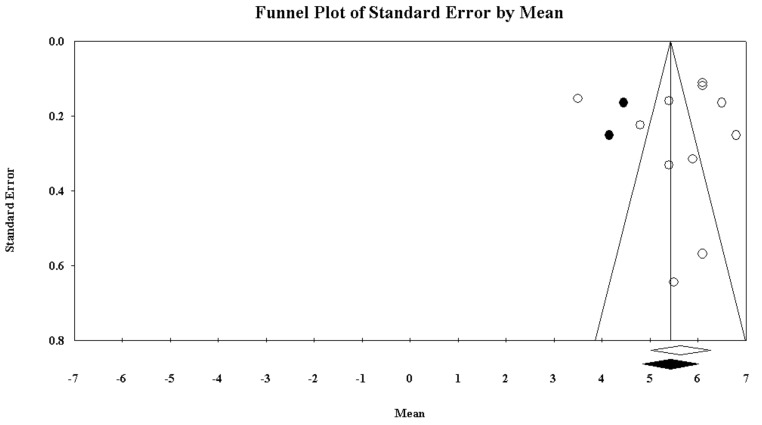
Funnel plot of the meta-analysis of the pooled PSQI global score in police officers.

The PSQI global score was reported by 11 papers and ranged between 6.8 and 3.5. In most of the researches included, a high PSQI global score was reported, well exceeding the PSQI cut-off of 5. As pictorially shown in Figure 6, the pooled mean score of the PSQI resulted 5.64 [95%CI 5.02–6.26]. Sensitivity analysis is shown in Figure 7, confirming the reliability of our findings. However, evidence of publication bias was detected (Figure 8). As such, the “true” ES was estimated to be 5.43 [95%CI 4.84–6.01], after trimming 2 studies.

**Figure 9:**
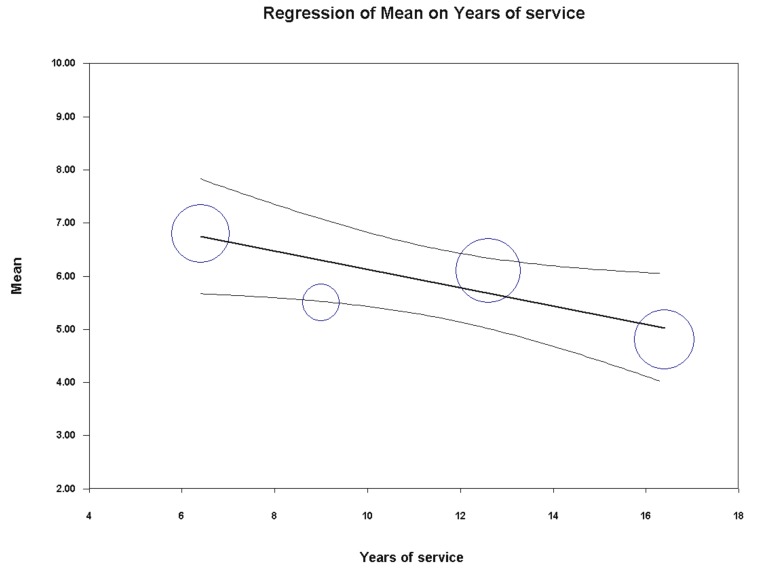
Meta-regression analysis showing a statistically significant association between the pooled PSQI global score in police officers and years of service.

**Figure 10:**
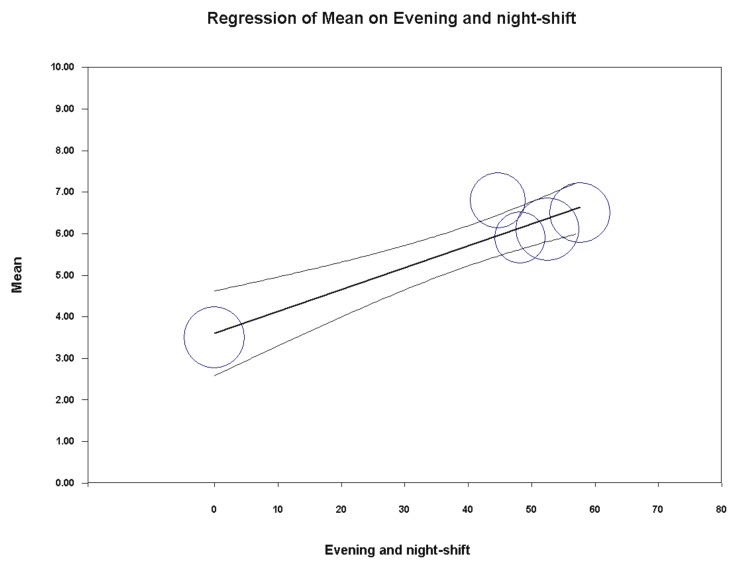
Meta-regression analysis showing a statistically significant association between the pooled PSQI global score in police officers and the percentage of policemen on evening/night shift.

Univariate meta-regression analysis indicated a significant association between the PSQI global score and years of service (coefficient = −0.17, SE = 0.07 [95%CI −0.31 to −0.04], z = −2.54, *p* = 0.0112) and percentage of policemen on evening/night shifts (coefficient = 0.05, SE = 0.01 [95%CI 0.03 to 0.08], z = 5.75, *p* = 0.0000) (Figure 9 and Figure 10). On the contrary, year of publication (*p* = 0.9902), country (*p* = 0.7936), gender (*p* = 0.4032), mean age (*p* = 0.2271), hierarchical role (*p* = 0.8845), BMI (*p* = 0.8689) and sample size (*p* = 0.3752) did not achieve the significance threshold. As for the previous meta-analysis, for the other variables under study, it was not possible to perform any meta-regression analysis due to the insufficient number of studies. Furthermore, for the same reason, it was not possible to run a multivariate meta-regression analysis.

Moreover, the main determinants of poor sleep quality were coded and grouped according to the main area/theme of interest. Some studies analyzed the association between sleep disorders and medical condition, such as metabolic syndrome, cardiovascular disease and blood pressure, or mental health, response to traumatic events, distress and depression. Other studies analyzed the association between sleep and quality of life, chronotype, shift work, and other occupational aspects. 

Shift work appears to be associated with sleep deprivation and bad sleep quality in police officers, especially if they are working "out-of-phase" with reference to chronotype.

Significant association between bad sleep quality and mental health was observed, even if, in the absence of longitudinal studies, it is not possible to know the direction of the association, i.e., whether it is the mental health status that determines sleep disorders, or the opposite. Chopko et al. [37], in a study on 193 police officers, argued that sleep quality mediates the association between post-traumatic stress disorder (PTSD) and health outcomes, including physical disorders or depression. Neylan et al. [43] observed that sleep disorders were strongly associated with post-traumatic stress symptoms and general psychopathology, but that critical incident exposure was only weakly associated with poor global sleep quality. In contrast, prolonged exposure to routine stress factors from officers’ general work environment was found to be strongly associated with poor global sleep quality. This result is in agreement with what is reported by other authors, according to which the stress of routine operations can be more harmful to the health of the police operators than exposure to a single stressful event. 

The study of Hartley et al. [41] on 356 police officers from the BCOPS cohort confirmed that different types of police stress may adversely affect sleep quality, and those with higher depressive symptoms may be more adversely affected by police stress. Everding et al. [39] observed that poor sleep was associated with decreased health-related quality of life and depressive symptoms. Chang et al. [35] showed that poor sleepers in Taiwanese police had a higher prevalence of metabolic syndrome (MetS) and abdominal obesity than other officers. In other studies, the association between quality of life and quality of sleep was statistically significant among military police officers. 

However, some scholars reported also negative findings. For example, Yoo and Franke [50] failed to demonstrate a significant association between sleep quality and MetS in a very small sample of police officers, even if they found a relationship between long sleep duration and MetS. Bernardo et al. [33] failed to demonstrate the association that would be expected between exercise/physical activity level and a good quality of sleep in a sample taken from the Brazilian military police.

## 4. Discussion

Our data documented that police officers show a considerable level of poor sleep quality as assessed by means of the PSQI, the most used tool to evaluate sleep disorders both in clinical and not clinical populations. The current quantitative analysis has allowed to estimate that more than half of policeman complain of bad sleep quality. Statistically significant associations were found with years of service and being on evening/night shift. More in detail, work seniority was found to correlate with higher sleep quality, suggesting that adaptive mechanisms and strategies could intervene, whereas shift work resulted in worse sleep outcomes. These results are highly relevant because police officers emerge as a population at augmented risk of sleep disorders. This characterizes police officers as population target for health promotion programs, especially focused on sleep hygiene and healthy lifestyle habits. These interventions could be particularly useful for police officers at the beginning of their career and for those on evening/night shift. To the best of our knowledge, this is the first systematic review and meta-analysis that provides a quantitative, updated estimation of subjective sleep quality among police officers. 

Sleep disorders are common but remain largely undiagnosed and untreated in general population, and also in police officers [51]. Rajaratnam et al. [25] performed the most comprehensive sleep disorders screening program in this occupational group and observed that 40.4% of police officers reported symptoms consistent with at least one sleep disorder, where the most prevalent disorder was obstructive sleep apnea (OSA), followed by moderate-to-severe insomnia and shift work disorder. Authors observed that sleep disorders were significantly associated with increased risk of self-reported adverse health, performance and safety outcomes. 

The quantitative synthesis conducted in the present study indicates that 51% of police officers reported bad sleep quality, with a pooled global PSQI score of 5.6. These data are significantly higher than those observed in community samples. The percentage of general population reporting poor sleep quality ranges between 26.5% in Austrian males and 32% in both genders [52] and 39% among adult Chinese in Hong Kong [53]. Hinz et al. [54] in a survey regarding a sample of more than 9,000 adults, obtained a PSQI global score of 4.38 for male participants. The results observed in our meta-analysis, obtained from observations composed mainly by men, confirmed that police officers present a significant higher PSQI score than the general population. 

The heterogeneity in our analysis was elevated. However, we could not perform a moderator analysis, because the determinants of sleep quality in the included studies were quite different. The possible reasons of the high heterogeneity could be the variety of samples employed in the studies by age, sex distribution, health conditions and others characteristics. Moreover, the low-moderate quality of the studies could have influenced data about the heterogeneity. Selected studies observed that occupational stress and traumatic events were among the strongest predictors of low sleep quality. These results are in line with those obtained among other workers and occupational groups. For instance, truck drivers that reported bad sleep quality had a worse psychological wellbeing than the group of good sleepers [55]. In a sample of firefighters, in addition to the high prevalence of sleep disturbance, a significant relationship between psychological distress and poor sleep quality was also identified [56]. 

Similar results were obtained in a wide sample of female workers in manufacturing jobs [57], and in a group of newspaper couriers who permanently woke up early [58]. Another important factor that could influence sleep quality is shift work. Police officer sleep and wellbeing could be affected by nightshift and long work schedules. This result confirms the findings observed in other categories of workers. 

Polysomnographic studies have demonstrated the existence of a dose-response relationship between the duration of shift work and the frequency of altered sleep patterns [58,59]. At the meta-regression analysis, we were able to find a statistically significant association and to confirm this relationship among police officers. The poor quality of sleep in the police officers of the selected studies was associated with physical illnesses, particularly of a cardiovascular nature, and poor mental health in the observed cohorts. The few observations that have studied the association of sleep disorders with adverse health effects do not allow to reach definitive evidence, but the results are in line with the literature trends. A recent longitudinal study observed that police officers suffering from stress and sleep disorders had a significantly greater risk of developing metabolic syndrome than their less distressed colleagues with good sleep habits [23]. 

In this review, all the selected studies had a cross-sectional design. This does not allow us to exclude reverse causation. The most plausible explanation of the observed association is that the poor quality of sleep could cause physical and mental illness, but the opposite relationship cannot be ruled out. A circular relationship between sleep and health variables is also possible. A recent review concluded that sleep can act as a moderator; therefore, a vicious circle between low wellbeing and poor sleep quality can affect the performance, the health and the safety of workers [60]. 

However, this systematic review and meta-analysis has some limitations. The exclusion of studies that did not use the PSQI may have reduced the number of observations and studies addressing sleep problems in police officers. However, this choice made it possible to compare very different cases with the same measure, ensuring the reliability of the findings. Another shortcoming is given by the medium-low quality of the included studies. This phenomenon is intrinsic to all the investigations conducted on the police forces. The most likely explanation is that the delicacy of police tasks discourages many researchers from tackling this sector with the due wealth of epidemiological methods. Finally, most data were from the United States. No studies carried out in Europe could be found. This could affect the generalizability of our results.

## 5. Conclusions

This study, the first meta-analysis about sleep quality in police officers, shows a high presence of poor sleep quality in this workers’ collective. The meta-analysis of the scores obtained with the PSQI in the different studies showed a pooled mean score higher than the cut-off that is considered indicative of poor quality of sleep. Significant associations with work seniority and shift work could be detected. 

Sleep disorders are a relevant issue among police officers, and can cause significant damage to health. Sleep health promotion programs are, therefore, strongly needed in police forces. The health and wellbeing of the police officers, because of the type of their work, should be pursued not only from the viewpoint of occupational medicine but also within the perspective of public health, in order to guarantee the safety of all of society. Society as a whole has a high interest in improving the health of police officers. The scientific world should contribute to this need by improving the quality of studies dedicated to police work.

## Figures and Tables

**Table 1:** Characteristics of the studies included in the present systematic review and meta-analysis.

Author (Year)	Type of Study, Quality	Sample Country)	Mean Age (% fem)	BMI Mean (SD)	Smoking Status (%)	Alcohol Intake	Married (%)	Caucasians and Hispanics (%)	Education (High–School %)	Years of Service (Work Hours per Day)	Hierarchical Role (%)	Administrative Role (%)	Military/Previous Military Experience	Evening and Night–Shift (%)	Prevalence of Poor Sleep Quality	PSQI Global Score (Number of Hours of Sleep)	Main Factor Related
Bernardo et al., (2018) [33]	Cross sec *	438 (Brazil)	33.2 ± 7.6 [20–53] (10.5%)	No data	No data	No data	No data	No data	No data	No data (no data)	4.6%	26.3%	Military police	73.7%	79.2%	No data (no data)	Physical activity (low, 20.5%)
Bond et al., (2013) [34]	Cross sec **	372 (USA)	41.3 ± 6.7 (27.7%)	29.1 ± 4.7	39.7%	5.6 ± 9.4	72.9%	79.3%	10.5%	14.6 ± 7.0 (high 63.8%)	16.6%	No data	22.3%	59.3%	54.8%	6.5 (6.1 ± 1.2)	Traumatic event exposure
Chang et al., (2015) [35]	Cross sec **	796 (China)	37.4 ± 7.7 [20–60] (0%)	25.2 ± 3.6	43.1%	1.8%	No data	No data	No data	No data	No data	No data	No data	52.5%	52.3%	6.1 ± 3.1 (6.1 ± 1.1)	Met S, shift work
Charles et al., (2011) [36]	Cross sec **	430 (USA)	42.1 ± 8.4 (25.8%)	29.2 ± 4.8	41.4%	5.6 ± 9.5	No data	80%	11%	No data (high 36.0%)	15.7%	No data	25.4%	57.6%	51.9%	No data (6.1 ± 1.2)	Perceived stress
Chopko et al. (2018) [37]	Cross sec *	193 (USA)	41.6 ± 9.2 [23–63] (6.7%)	No data	No data	No data	82.4%	93.3%	14.7 ± 1.9 years [12–22]	16.4 ± 8.9 [1–42]	15%	No data	No data	No data	No data	4.8 ± 3.1 (no data)	PTSD, general health
Elliot et al., (2016) [38]	Cross sec *	206 (Australia)	31.6 ± 8.5 (32%)	No data	No data	No data	No data	No data	No data	6.4 ± 7.5	17.5%	No data	No data	44.7%	69%	6.8 ± 3.6(no data)	Blood pres., fatigue, card. diseases
Everding et al., (2016) [39]	Cross sec *	379 (USA)	41.5 ± 8.6 (6%)	28.8 ± 3.9	No data	No data	No data	No data	No data	No data	No data	No data	No data	No data	38%	5.4 ± 3.1 (no data)	Card. disease and mental health, inflammatory markers
Fekedulegn et al., (2016) [40]	Cross sec **	363 (USA)	41.2 ± 6.6 [27–66] (28%)	29.2 ± 4.7 (BMI>25 81%)	39.3%	5.5 ± 9.4	72.2%	76.5%	10.5%	14.4 ± 6.8 (high 64%)	28.1%	No data	No data	50.4%	54%	No data (no data)	Shift work
Hartley et al., (2014) [41]	Cross sec **	356 (USA)	41.3 ± 6.7 (28%)	29.2 ± 4.7	40.4%	5.6 ± 9.4	73.9%	79.5%	10.7%	49% ≥15 years (no data)	28.1%	No data	21.4%	59.9%	54.2%	6.5 ± 3.4 (6.1 ± 1.2)	Stress
McCanlies et al., (2012) [42]	Cross sec **	98 (USA)	39.6 (39.8%)	No data	53.1%	No data	65.3%	81.6%	18.4%	No data	34.7%	No data	No data	No data	No data	No data (6.4)	Met S
Neylan et al., (2002) [43]	Cross sec **	733 (USA)	37.0 (29%)	No data	No data	No data	68.8&	69%	28%	12.6 (no data)	No data	No data	No data	No data	64%	6.1 ± 3.2 *	Critical incidents
Neylan et al., (2010) [44]	Cross sec *	189 (USA)	26.4 ± 4.2 [21–43] (12%)	No data	No data	No data	No data	No data	No data	No data	No data	No data	No data	0%	No data	3.5 ± 2.1 (6.25 ± 1.70)	Psychomotor performance
Pinto et al., (2018) [45]	Cross sec *	22 (Brazil)	34.6 ± 6.1 (0%)	25.2 [23–31]	No data	No data	No data	No data	22.7%	9 [6–25] (No data)	27.2%	No data	Military	No data	63.6%	5.5 ± 3.0 *(6.5 ± 0.8)	QoL, work accidents
Ramey et al., (2012) [46]	Cross sec *	85 (USA)	39.6 ± 9.0 [22.4–63.3] (0%)	80%> 25	No data	No data	No data	No data	No data	No data (45.9 ± 7.5 [8–70])	No data	No data	No data	41/85 (48.2%)	23%	5.9 ± 2.9 [2–13] (6.8 ± 1.3 [3.5–9.5])	Shift work
Roustaei et al., (2017) [47]	Cross sec *	60 (Iran)	No data (100%)	No data	No data	No data	No data	No data	No data	No data	No data	50%	No data	No data	48.3%	6.1 (no data)	QoL
Slaven et al., (2011) [48]	Cross sec **	391 (USA)	40.7 ± 7.1 (27.4%)	No data	40%	5.4 ± 9.2	73%	80%	10%	No data	No data	No data	No data	No data	66.2%	6.5 ± 3.4 (no data)	Depression
Yadav et al., (2016) [49]	Cross sec *	85 (India)	26.6 ± 0.6 (60%)	22.9 ± 0.2	No data	No data	No data	No data	No data	No data	No data	No data	No data	No data	31.8%	No data (no data)	Chronotype and duty schedule
Yoo et al., (2013) [50]	Cross sec *	106 (USA)	42.3 ± 8.4 (0%)	29.7 ± 4.1	No data	No data	No data	No data	No data	No data	No data	No data	No data	No data	36.8%	5.4 ± 3.4 (no data)	Met S, mental health

Notes: *: low study quality; **: medium study quality. Abbreviations: BMI (body mass index); Met S: metabolic syndrome; PSQI: Pittsburgh Sleep Quality Index; PTSD: post-traumatic stress disorder; QoL: quality of life; SD (standard deviation).
